# A Facile, Improved Synthesis of Sildenafil and Its Analogues

**DOI:** 10.3390/scipharm84030447

**Published:** 2015-10-18

**Authors:** Ambati. V. Raghava Reddy, Garaga Srinivas, Chandiran Takshinamoorthy, Badarinadh Gupta Peruri, Andra Naidu

**Affiliations:** 1 Aurobindo Pharma Limited Research Centre-II, Survey No. 71&72, Indrakaran Village, Sangareddy Mandal, Medak District 502329, Telangana, India; srinivasgaraga@yahoo.com (G.S.); chandiran.t@aurobindo.com (C.T.); badari_peruri@yahoo.com (B.G.P.); 2 Department of Chemistry, Jawaharlal Nehru Technological University Hyderabad, Kukatpally, Hyderabad 500085, Telangana, India; andranaidu@gmail.com

**Keywords:** sildenafil, chlorosulfonation, novel analogues

## Abstract

This paper describes the facile synthesis of sildenafil citrate (**1**) and its related compounds through an improved chlorosulfonation reaction using chlorosulfonic acid and thionyl chloride. This synthesis results in pure sildenafil with high yield. The structures of the compounds were determined with infrared (IR), ^1^H-NMR and ^13^C-NMR spectroscopic methods, and high resolution mass spectroscopy (HRMS) analysis.

## 1. Introduction

Sildenafil citrate (**1**) is a selective inhibitor of cyclic guanosine monophosphate (cGMP)-specific phosphodiesterase type 5 (PDE5), which is responsible for the degradation of cGMP in the corpus cavernosum.

**Figure 1:**
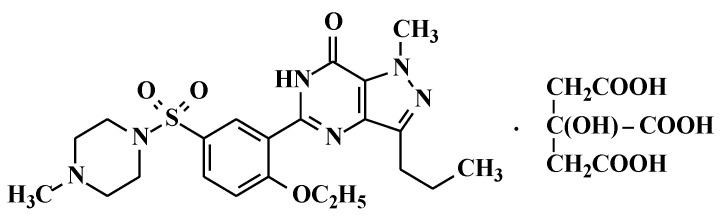
Structure of sildenafil citrate (**1**).

**Scheme 1:**
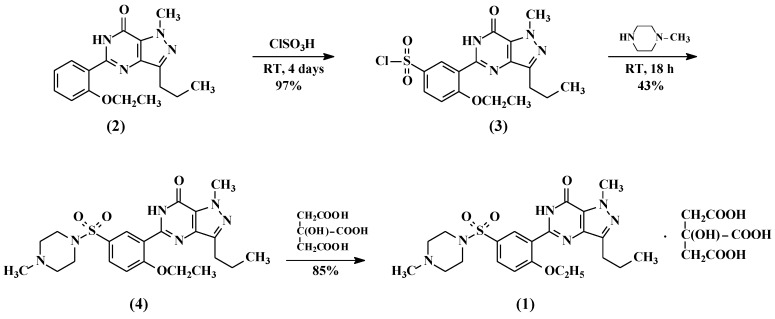
Reported route for synthesis of sildenafil citrate.

Sildenafil is used in the treatment of erectile dysfunction in males and it is also used for hypertension [1,2]. It is chemically known as 5-{2-ethoxy-5-[(4-methylpiperazinyl)sulfonyl] phenyl}-1-methyl-3-*n*-propyl-1,6-dihydro-7*H*-pyrazolo[4,3-*d*]pyrimidin-7-one-2-hydroxy-1,2,3-propanetricarboxylate. As per the first process disclosed in the literature [3], sildenafil (**4**) was prepared by reacting 5-(2-ethoxyphenyl)-1-methyl-3-*n*-propyl-1,6-dihydro-7*H*-pyrazolo[4,3-*d*]pyrimid-7-one (**2**) with chlorosulfonic acid to produce 5-(5-chlorosulfonyl-2-ethoxyphenyl)-1-methyl-3-*n*-propyl-1,6-dihydro-7*H*-pyrazolo[4,3-*d*] pyrimidin-7-one (**3**), which was further converted into sildenafil by reacting with *N*-methyl piperazine. Finally, poorly water soluble Sildenafil (**4**) is converted in to Sildenafil citrate (**1**) (Figure 1) by treating with citric acid monohydrate to improve its solubility and bioavailability (Scheme 1).

**Scheme 2:**
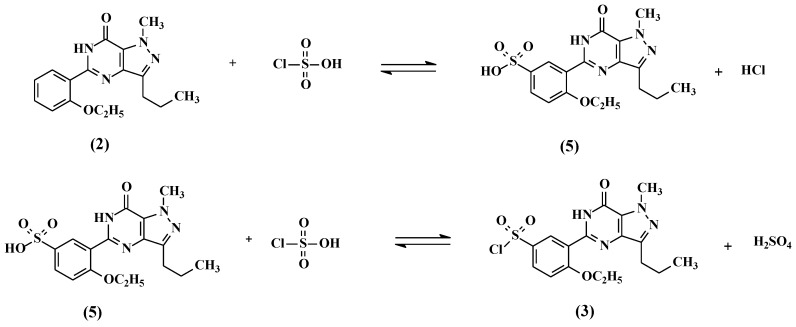
Synthetic path of chlorosulfonation.

In the above reported synthesis, preparation of compound **3** from compound **2** was critical as it highly influenced the yield and quality of the end product. The chlorination of compound **5** was a reversible reaction (Scheme 2, [4]), so the experimental conditions had to be adjusted to obtain the maximum conversion of compound **2** into sulfonyl chloride (compound **3**).

## 2. Materials and Methods

**General Procedures:** All melting points were determined with the Polmon melting point apparatus(Polmon Instruments Pvt. Ltd., Hyderabad, India). ^1^H-NMR and ^13^C-NMR spectra were recorded on a Bruker 300 & 500 spectrometer (Bruker, Fällanden, Switzerland). Chemical shifts (δ) were reported in ppmdownfield with TMS as internal standard, multiplicities are described as s: singlet, d: doublet, t: triplet, dd: double doublet, m: multiplet, brs: broad singlet. The HRMS spectra were measured on the Perkin Elmer PE SCIEX-API 2000 mass spectrometer (Waters, MA, United States). An analytical HPLC (Waters, MA, United States) was run with the Symmetry C_18_, 210 × 4.6 mm column at 290 nm.

### 2.1. Preparation of 5-(5-Chlorosulfonyl-2-ethoxyphenyl)-1-methyl-3-n-propyl-1,6-dihydro-7H-pyrazolo[4,3-d]pyrimidin-7-one (***3***)

5-(2-ethoxyphenyl)-1-methyl-3-*n*-propyl-1,6-dihydro-7*H*-pyrazolo[4,3-*d*]pyrimid-7-one (**2**, 25 g, 80.13 mmol) followed by thionyl chloride (9.53 g, 80.13 mmol) were added to chlorosulfonic acid (50 mL) portion-wise at 0–10 °C. The reaction mass temperature was raised to 20–30 °C and stirred for 4 h to complete the reaction. The reaction mass was poured onto ice (~500 g) slowly and the product was extracted with dichloromethane (250 mL). The dichloromethane layer was separated and washed with 5% w/w aqueous sodium bicarbonate (100 mL). The dichloromethane layer containing compound **3** was taken to the next step as such.

### 2.2. Preparation of 5-[2-Ethoxy-5-(4-methylpiperazinylsulfonyl)phenyl]-1-methyl-3-n-propyl-1,6-dihydro-7H-pyrazolo[4,3-d]pyrimidin-7-one (Sildenafil, ***4***)

*N*-methylpiperazine (9.6 g, 96 mmol) was added to the dichloromethane layer containing sulfonyl chloride compound **3** (obtained from example **1**) and stirred for 1 h at 20–25 °C. Then the reaction mass was washed with 5% w/w aqueous sodium bicarbonate (100 mL) followed by demineralised (DM) water (100 mL). The dichloromethane layer was concentrated at <50 °C and methanol was added to the residue to crystallize the product. The product was filtered and dried at 55–60 °C under vacuum to obtain 34 g of pure sildenafil (90% yield).

### 2.3. General Procedure for the Preparation of Compounds ***10a***–***10c***

To a mixture of diethyl oxalate (100 g, 685 mmol) and appropriate ketone (685 mmol), powdered sodium methoxide (37.8 g, 685 mmol) was added in portions at 20 °C. The reaction was heated to 50–55 °C and stirred for 1 h, the reaction was cooled to 20–25 °C, then diluted with ethyl acetate (200 mL) and DM water (200 mL). Further, the pH was adjusted to 1.8 with diluted hydrochloric acid and separated into two layers. The ethyl acetate layer was washed with 20% aqueous sodium chloride and dried over anhydrous sodium sulfate. The ethyl acetate layer was cooled to ~10 °C and *para*-toluensulfonic acid (2.96 g, 15.58 mmol) was added followed by hydrazine hydrate (30 mL, 80%) over a period of 2 h at 10–15 °C. The reaction mass was stirred for 1 h and diluted with water (200 mL). The pH of the reaction mass was adjusted to 7.2 with 30% aqueous sodium hydroxide and the layers were separated. The bottom aqueous layer was extracted with ethyl acetate (100 mL) and mixed with the first ethyl acetate layer. The combined ethyl acetate layers were concentrated at 40–45 °C under reduced pressure to obtain the title compounds **7a**–**7c** as an oily residue. 

The residue was cooled to 20–25 °C and dimethyl sulphate (86.31 g, 685 mmol) was added in 2 h at 20–25 °C. The temperature was raised to 55–60 °C and maintained for 2 h. The reaction mixture was cooled to 10–15 °C and poured into a mixture of dichloromethane (500 mL) and DM water (500 mL). The pH of the reaction was adjusted to 7.2 with 30% aqueous sodium hydroxide and the two layers were separated. The organic layer was concentrated at 40–45 °C to obtain compounds **9a**–**9c** as an oily mass. Five N aqueous sodium hydroxide (200 mL, 1 mol) was added to the oily residue and the mixture was heated to 80 °C for 2 h. The reaction temperature was brought down to 25–30 °C and the pH was adjusted to ~1.0 with hydrochloric acid. The solid precipitate was collected by filtration, washed with water, and dried to obtain the carboxylic acid compounds **10a**–**10c**. 

*Preparation of 3-ethyl-1-methyl-1H-pyrazole-5-carboxylic acid* (**10a**). Compound **10a** was prepared from 2-butanone as a pale yellow solid. Yield 53%, ^1^H-NMR (DMSO-*d*_6_) δ: 1.131–1.182 (t, 3H, CH_3_), 2.510–2.549 (m, 2H, CH_2_), 3.759 (s, 3H, CH_3_), 6.91 (s, 1H, COOH). Mass: 155.217 (M+H)^+^.

*Preparation of 3-butyl-1-methyl-1H-pyrazole-5-carboxylic acid* (**10b**). Compound **10b** was prepared from 2-hexanone as a pale yellow solid. Yield 55%, ^1^H-NMR (DMSO-*d*_6_) δ: 0.86–0.91 (q, 3H, CH_3_), 1.27–1.34 (m, 2H, CH_2_), 1.50–1.57 (m, 2H, CH_2_), 2.49–2.54 (t, 2H, CH_2_), 3.99 (s, 3H, *N*-CH_3_), 6.60 (s, 1H, CH), 13.18 (bs, 1H, COOH). Mass: 183.22 (M + H)^+^.

*Preparation of 3-isopropyl-1-methyl-1H-pyrazole-5-carboxylic acid* (**10c**). Compound **10c** was prepared from 3-methyl-2-butanone as a pale yellow solid. Yield 56%, ^1^H-NMR (DMSO-*d*_6_) δ: 1.17–1.20 (m, 6H, 2xCH_3_), 2.80–2.92 (m, 1H, CH(CH_3_)_2_), 4.0 (s, 3H, *N*-CH_3_), 6.63 (s, 1H, COOH). Mass: 169.27 (M + H)^+^.

### 2.4. General Procedure for the Preparation of Compounds ***11a***–***11c***

Sodium nitrate (380 mmol) was slowly added to sulfuric acid (192 mL) in about 30 min at 0–5 °C. The contents were heated to 25–30 °C and stirred for 1 h. Thereafter, carboxylic acid (**10a**–**10c**, 380 mmol) was added portion–wise, keeping the temperature below 40 °C. After the addition, the reaction was heated at 60 °C and maintained for 18 h. The reaction was cooled to room temperature before it was poured onto ice, then filtration and drying gave 4-nitrocarboxylic acid (**11a**–**11c**) as a white solid.

*Preparation of 3-ethyl-1-methyl-4-nitro-1H-pyrazole-5-carboxylic acid* (**11a**). Compound **11a** was prepared from **10a** as a white solid. Yield 82%, ^1^H-NMR (DMSO-*d*_6_) δ: 1.17–1.26 (t, 3H, CH_3_), 2.807–2.857 (m, 2H, CH_2_), 3.894 (s, 3H, *N*-CH_3_). Mass: 200.27 (M + H)^+^.

*Preparation of 3-butyl-methyl-4-nitro-1H-pyrazole-5-carboxylic acid* (**11b**). Compound **11b** was prepared from **10b** as a white solid. Yield 94%, ^1^H-NMR (DMSO-*d*_6_) δ: 0.874–0.966 (m, 3H, CH_3_), 1.328–1.397 (m, 2H, CH_2_), 1.546–1.646 (m, 2H, CH_2_), 2.766–2.817 (t, 2H, CH_2_), 3.912 (s, 3H, *N*-CH_3_). Mass: 228.22 (M + H)^+^.

*Preparation of 3-isopropyl-1-methyl-4-nitro-1H-pyrazole-5-carboxylic acid* (**11c**). Compound **11c** was prepared from **10c** as a white solid. Yield 81%, ^1^H-NMR (DMSO-*d*_6_) δ: 1.23–1.29 (m, 6H, 2CH_3_), 3.32–3.41 (m, 1H, CH(CH_3_)_2_), 3.90 (s, 3H, *N*-CH_3_). Mass: 214.19 (M + H)^+^.

### 2.5. General Procedure for the Preparation of Compounds ***12a***–***12c***

4-Nitrocarboxylic acid (**11a**–**11c**, 352 mmol) was added to thionyl chloride (200 mL) and the resulting mixture was heated under reflux for 3 h. Thereafter, excess thionyl chloride was removed by evaporation under vacuum. The oily residue was dissolved in acetone (750 mL) and the pH was adjusted to 10 with aqueous ammonia. Acetone was distilled off and the mass was diluted by adding DM water (750 mL). The resulting slurry was filtered and washed with DM water to provide 4-nitrocarboxamide (**12a**–**12c**) as a pale yellow solid.

*Preparation of 3-ethyl-1-methyl-4-nitro-1-H-pyrazole-5-carboxamide* (**12a**). Compound **12a** was prepared from compound **11a** as a pale yellow solid. Yield 92%, ^1^H-NMR (DMSO-*d*_6_) δ: 1.045–1.083 (t, 3H, CH_3_), 2.359–2.456 (q, 2H, CH_2_), 3.92 (s, 3H, *N*-CH_3_), 7.483–7.695 (brs, 2H, NH_2_). Mass: 199.18 (M + H)^+^.

*Preparation of 3-butyl-1-methyl-4-nitro-1H-pyrazole-5-carboxamide* (**12b**). Compound **12b** was prepared from compound **11b** as a pale yellow solid. Yield 90%, ^1^H-NMR (DMSO-*d*_6_) δ: 0.88–0.93 (t, 3H, CH_3_), 1.27–1.39 (m, 2H, CH_2_), 1.55–1.65 (m, 2H, CH_2_), 2.80–2.85 (t, 2H, CH_2_), 3.78 (s, 3H, *N*-CH_3_), 8.23, 8.42 (2brs, 2H, CONH_2_). Mass: 227.3 (M + H)^+^.

*Preparation of 3-isopropyl-1-methyl-4-nitro-1H-pyrazoIe-5-carboxamide* (**12c**). Compound **12c** was prepared from compound **11c** as a pale yellow solid. Yield 90%, ^1^H-NMR (DMSO-*d*_6_) δ: 1.23–1.26 (m, 6H, 2xCH_3_), 3.34–3.51 (m, 1H, CH(CH_3_)_2_), 3.79 (s, 3H, *N*-CH_3_), 8.23, 8.43 (2brs, 2H, CONH_2_). Mass: 213.21 (M + H)^+^.

### 2.6. General Procedure for the Preparation of Compounds ***13a***–***13c***

A solution of 4-nitro carboxamide (**12a**–**12c**, 94 mmol) in methanol (400 mL) and 5% Pd/C (5 g, 50% wt) was charged into a one-litre hydrogenator at 25–30 °C. The reaction mixture was hydrogenated with 4 kg/cm^2^ pressure at 25–30 °C for ~5 h. After completion of the reaction, the hydrogen pressure was released and carefully filtered to remove the catalyst under the nitrogen atmosphere. The filtrate was concentrated under reduced pressure at 40–45 °C and the product was crystallized by adding ethyl acetate (250 mL). The product was filtered and dried to obtain the 4-aminocarboxamide compound (**13a**–**13c**).

*Preparation of 3-ethyl-1-methyl-4-amino-1-H-pyrazole-5-carboxamide* (**13a**). Compound **13a** was prepared from compound **12a**. Yield 84%, ^1^H-NMR (DMSO-*d*_6_) δ: 1.055–1.108 (t, 3H, CH_3_), 2.359–2.456 (q, 2H, CH_2_), 3.994 (s, 3H, *N*-CH_3_), 7.483–7.695 (brs, 2H, NH_2_). Mass: 169.20 (M + H)^+^.

*Preparation of 3-butyl-1-methyl-4-amino-1-H-pyrazole-5-carboxamide* (**13b**). Compound **13b** was prepared from compound **12b**. Yield 82%, ^1^H-NMR (DMSO-*d*_6_) δ: 0.86–0.93 (m, 3H, CH_3_), 1.27–1.35 (m, 2H, CH_2_), 1.48–1.53 (m, 2H, CH_2_), 2.42–2.50 (m, 2H, CH_2_), 3.85 (s, 3H, *N*-CH_3_), 4.09 (brs, 2H, NH_2_), 7.48 (brs, 2H, CONH_2_). Mass: 197.25 (M + H)^+^.

*Preparation of 3-isopropyl-1-methyl-4-amino-1-H-pyrazole-5-carboxamide* (**13c**). Compound **13c** was prepared from compound **12c**. Yield 81%, ^1^H-NMR (DMSO-*d*_6_) δ: 1.10–1.17 (m, 6H, 2CH_3_), 2.92–3.02 (m, 1H, CH(CH_3_)_2_), 3.94 (s, 3H, *N*-CH_3_) 4.08 (brs, 2H, NH_2_), 7.50 (brs, 2H, CONH_2_). Mass: 183.1 (M + H)^+^.

### 2.7. General Procedure for the Preparation of Compounds ***14a***–***14c***

To a solution of 4-amino carboxamide (**13a**–**13c**, 50 mmol) and triethylamine (10.12 g, 100 mmol) in dichloromethane (250 mL), 2-ethoxybenzoyl chloride (10 g, 54.5 mmol) was added at 0–5 °C. The resulting mixture was allowed to warm to room temperature and it was stirred for a further 2 h. The reaction mixture was washed with DM water twice and the organic layer was concentrated under reduced pressure. Then *n*-hexane (250 mL) was added to the residue and stirred for 1 h to precipitate the product. The product was collected by filtration and dried to obtain pyrazole carboxamide, compounds (**14a**–**14c**).

*Preparation of 3-ethyl-4-(2-ethoxybenzamido)-1-methyl-1H-pyrazole-5-carboxamide* (**14a**). Compound **14a** was prepared from compound **13a**. Yield 74%, ^1^H-NMR (DMSO-*d*_6_) δ: 1.11–1.16 (t, 3H, CH_2_CH_3_), 1.37–1.41 (t, 3H, OCH_2_CH_3_), 2.45–2.51 (q, 2H, CH_2_CH_3_), 3.92 (s, 3H, *N*-CH_3_), 4.17–4.23 (q, 2H, OCH_2_CH_3_), 7.03–7.08 (t, 1H, Ar), 7.16–7.19 (d, 1H, Ar), 7.47–7.53 (m, 1H, Ar), 7.64–7.67 (m, 1H, Ar), 7.37 & 7.76 (2brs,2H, CONH_2_), 9.49 (s, 1H, -CONH)*.* Mass: 317.35 (M + H)^+^.

*Preparation of 3-butyl-4-(2-ethoxybenzamido)-1-methyl-1H-pyrazole-5-carboxamide* (**14b**). Compound **14b** was prepared from compound **13b**. Yield 83%, ^1^H-NMR (DMSO-*d*_6_) δ: 0.83–0.88 (t, 3H, CH_2_CH_3_), 1.27–1.41 (t, 5H, CH_2_ & CH_3_), 1.52–1.57 (m, 2H, CH_2_), 2.44–2.51 (m, 2H, OCH_2_), 3.92 (s, 3H, *N*-CH_3_), 4.17–4.24 (q, 2H, OCH_2_), 7.03–7.08 (m, 1H, Ar), 7.17–7.19 (dd, 1H, Ar), 7.47–7.53 (m, 1H, Ar), 7.63 & 7.66 (m, 1H, Ar), 7.36 & 7.76 (2brs, 2H, CONH_2_), 9.48 (s, 1H, CONH). Mass: 345.31 (M + H)^+^.

*Preparation of 3-isopropyl-4-(2-ethoxybenzamido)-1-methyl-1H-pyrazole-5-carboxamide* (**14c**). Compound **14c** was prepared from compound **13c**. Yield 81%, ^1^H-NMR (DMSO-*d*_6_) δ: 1.14–1.22 (m, 6H, CH_3_), 1.37–1.41 (t, 3H, CH_3_), 2.88–2.97 (m, 1H, CH(CH_3_)_2_), 3.92 (s, 3H, *N*-CH_3_), 4.17–4.24 (q, 2H, OCH_2_CH_3_), 7.03–7.08 (m, 1H, Ar), 7.17–7.19 (dd, 1H, Ar), 7.47–7.53 (m, 1H, Ar), 7.63–7.66 (m, 1H, Ar), 7.33 & 7.74 (2brs, 2H, CONH_2_), 9.45 (s, 1H, CONH). Mass: 331.42 (M + H)^+^.

### 2.8. General Procedure for the Preparation of Compounds ***2a***–***2c***

Pyrazole carboxamide compound (**14a**–**14c**, 42.42 mmol) was added portion-wise to a solution of sodium hydroxide (3.4 g, 85 mmol) and 30% hydrogen peroxide solution (10 mL) in DM water (200 mL). Ethanol (50 mL) was added and the resulting mixture was heated under reflux for 8 h. Thereafter, the reaction mixture was cooled and evaporated under vacuum. The resulting solid was treated with 2 N hydrochloric acid (100 mL) at 25–30 °C and the mixture was extracted with dichloromethane (2 × 200 mL). The combined organic extracts were washed successively with saturated aqueous sodium carbonate (100 mL) and saturated sodium chloride solution (100 mL). The dichloromethane layer was concentrated and *n*-hexane was added to the residue to crystallize the product. The product was collected by filtration and dried to obtain the cyclized compounds **2a**–**2c**.

*Preparation of 3-ethyl-5-(2-ethoxyphenyl)-1-methyl-7H-pyrazolo[4,3-d]pyrimidin-7-one* (**2a**). Compound **2a** was prepared from compound **14a** as a white solid. Yield 61%, ^1^H-NMR (DMSO-*d*_6_) δ: 1.26–1.35 (m, 6H, 2CH_3_), 2.78–2.86 (q, 2H, CH_2_), 4.09–4.14 (q, 2H, OCH_2_), 4.15 (s, 3H, *N*-CH_3_), 7.04–7.09 (t, 1H, Ar), 7.14–7.16 (dd, 1H, Ar), 7.46–7.51 (m, 1H, Ar), 7.65–7.68 (t, 1H, Ar), 11.97 (s, 1H, NH). Mass: 299.1 (M + H)^+^.

*Preparation of 3-butyl-5-(2-ethoxyphenyl)-1-methyl-7H-pyrazolo[4,3-d]pyrimidin-7-one* (**2b**). Compound **2b** was prepared from compound **14b** as a white solid. Yield 65%, ^1^H-NMR (DMSO-*d*_6_) δ: 0.88–0.93 (t, 3H, CH_2_CH_3_), 1.31–1.39 (m, 5H, CH_2_ & CH_3_), 1.68–1.73 (m, 2H, CH_2_), 2.77–2.82 (t, 2H, CH_2_), 4.09–4.12 (m, 2H, OCH_2_), 4.15 (s, 3H, *N*-CH_3_), 7.04–7.09 (t, 1H, Ar), 7.14–7.16 (dd, 1H, Ar), 7.45–7.51 (m, 1H, Ar), 7.64, 7.66 (m, 1H, Ar), 11.95(s, 1H, -NH), 9.48 (s, 1H, -CONH). Mass: 327.2 (M + H)^+^.

*Preparation of 3-isopropyl-5-(2-ethoxyphenyl)-1-methyl-7H-pyrazolo[4,3-d]pyrimidin-7H-one* (**2c**): Compound **2c** was prepared from compound **14c** as a white solid. Yield 71%, ^1^H-NMR (DMSO-*d*_6_) δ: 1.31–1.36 (m, 9H, 3xCH_3_), 3.34–3.42 (m, 1H, CH(CH_3_)_2_), 4.09–4.15 (q, 2H, OCH_2_), 4.16 (s, 3H, *N*-CH_3_), 7.04–7.09 (t, 1H, Ar), 7.14–7.16 (dd, 1H, Ar), 7.45–7.50 (m, 1H, Ar), 7.65–7.68 (m, 1H, Ar), 11.95 (s, 1H, NH). Mass: 313.1 (M + H)^+^.

### 2.9. General Procedure for the Preparation of Compounds ***3a***–***3c***

Cyclized compounds (**2a**–**2c**, 80.13 mmol) followed by thionyl chloride (9.53 g, 80.13 mmol) were added to chlorosulfonic acid (50 mL) portion-wise at 0–10 °C. The reaction temperature was raised to 20–30 °C and stirred for 4 h. The reaction mass was poured onto ice (500 g) and extracted with dichloromethane (250 mL). The dichloromethane layer was separated and washed with 5% w/w aqueous sodium bicarbonate (100 mL). The dichloromethane layer containing compound **3** was taken to next step as such.

*Preparation of 5-(5-chlorosulfonyl-2-ethoxyphenyl)-1-methyl-3-ethyl-1,6-dihydro-7H-pyrazolo[4,3-d]pyrimidin-7-one* (**3a**). Compound **3a** was prepared from compound **2a**. Dichloromethane solution containing compound **3a** was used as such in the next step.

*Preparation of 5-(5-chlorosulfonyl-2-ethoxyphenyl)-1-methyl-3-butyl-1,6-dihydro-7H-pyrazolo[4,3-d]pyrimidin-7-one* (**3b**). Compound **3b** was prepared from compound **2b**. Dichloromethane solution containing compound **3b** was used as such in the next step.

*Preparation of 5-(5-chlorosulfonyl-2-ethoxyphenyl)-1-methyl-3-isopropyl-1,6-dihydro-7H-pyrazolo[4,3-d]pyrimidin-7-one* (**3c**). Compound **3c** was prepared from compound **2c**. Dichloromethane solution containing compound **3c** was used as such in the next step.

### 2.10. General Procedure for the Preparation of Compounds **4a**–**4c**

*N*-methylpiperazine (9.6 g, 96.15mmol) was added to the dichloromethane layer, containing sulfonyl chloride (**3a**–**3c**, obtained from example **9**) at 20–25 °C. The reaction was stirred for 4 h and then washed with 5% w/w aqueous sodium bicarbonate (100 mL) followed by DM water (100 mL). The dichloromethane layer was concentrated at <40 °C under reduced pressure to obtain a foamy material. Methanol was added to crystallize the product and it was dried to result in compounds **4a**–**4c**.

*Preparation of 5-[2-ethoxy-5-(4-methylpiperazinylsulfonyl)phenyl]-1-methyl-3-ethyl-1,6-dihydro-7H-pyrazolo[4,3-d]pyrimidin-7-one* (sildenafil ethyl analogue, **4a**). Sildenafil ethyl analogue (**4a**) was prepared from compound **3a**. Yield: 91%, Melting point: 203–206 °C, HRMS for C_21_H_28_N_6_O_4_S (M + H)^+^ calc.: 460.1892, found: 460.1912, ^1^H-NMR (DMSO-*d*_6_) δ: 1.26–1.35 (m, 6H, 2CH_3_), 2.14 (s, 3H, CH_3_), 2.36 (s, 4H, 2xCH_2_ of piperazine), 2.79–2.86 (m, 2H, CH_2_, of piperazine), 2.90 (s, 4H, 2xCH_2_, of piperazine), 4.16 (s, 3H, *N*-CH_3_), 4.20–4.25 (q, 2H, OCH_2_), 7.36–7.39 (d, *J* = 9 Hz, 1H, Ar), 7.81–7.85 (t, *J* = 9.3 Hz, 2H, Ar),12.21 (brs, 1H, NH). ^13^C-NMR (DMSO-*d*_6_) δ:13.1, 14.2, 18.6, 37.8, 38.7, 39.2, 39.8, 40.3, 45.3, 45.7, 53.5, 64.8, 113.2, 123.7, 124.5, 126.2, 129.9, 131.4, 137.4, 146.1, 148.1, 153.7, 159.9. IR (KBr pellet): 3104, 2976, 2946, 2802, 1690, 1598, 1456, 1348, 1287, 1171 cm^−1^.

*Preparation of 5-[2-ethoxy-5-(4-methylpiperazinylsulfonyl)phenyl]-1-methyl-3-n-butyl-1,6-dihydro-7H-pyrazolo[4,3-d]pyrimidin-7-one* (sildenafil butyl analogue, **4b**). Sildenafil butyl analogue (**4b**) was prepared from compound **3b**. Yield: 90%, Melting point: 189–192 °C, HRMS for C_23_H_32_N_6_O_4_S (M + H)^+^ calc.: 488.2205, found: 488.2310, ^1^H-NMR (DMSO-*d*_6_) δ: 0.88–0.93 (t, *J* = 9,6 Hz, 3H, CH_3_), 1.31–1.39 (m, 5H, CH_2_& CH_3_), 1.70 (m, 2H, CH_2_ of piperazine), 2.14 (s, 3H, CH_3_), 2.36 (s, 4H, 2CH_2_, of piperazine), 2.50–2.80 (m, 2H, -CH_2_), 2.90 (s, 4H, 2CH_2_ of piperazine), 4.16 (s, 3H, *N*-CH_3_), 4.20–4.23 (q, 2H, -OCH_2_), 7.36–7.39 (d, *J* = 9 Hz, 1H, Ar), 7.81–7.85 (t, *J* = 12 Hz, 2H, Ar),12.19 (brs, 1H, -NH).^13^C-NMR (DMSO-*d*_6_) δ:13.6, 14.2, 21.8, 24.7, 30.5, 37.8, 38.9, 39.2, 39.5, 39.8, 40.1, 40.3, 45.3, 45.7, 53.5, 64.8, 113.2, 123.7, 124.4, 126.2, 129.9, 131.5, 137.7, 145.1, 148.1, 153.7, 159.9. IR (KBr pellet): 3107, 2959, 2937, 2796, 1689, 1599, 1488, 1349, 1288, 1171 cm^−1^.

*Preparation of 5-[2-ethoxy-5-(4-methylpiperazinylsulfonyl)phenyl]-1-methyl-3-isopropyl-1,6-dihydro-7H-pyrazolo[4,3-d]pyrimidin-7-one* (sildenafil isopropyl analogue, **4c**). Sildenafil isopropyl analogue (**4c**) was prepared from compound **3c**. Yield: 90%, Melting point: 212–215 °C, HRMS for C_22_H_30_N_6_O_4_S (M + H)^+^ calc.: 474.2049, found: 474.2105, ^1^H-NMR (DMSO-*d*_6_) δ: 1.31–1.36 (m, 9H, -3xCH_3_), 2.14 (s, 3H, -CH_3_), 2.36 (s, 4H, 2CH_2_ of piperazine), 2.91 (s, 4H, 2CH_2_, of piperazine), 4.16 (s, 3H, -NCH_3_), 4.18–4.23 (q, 2H, -OCH_2_), 7.36–7.39 (d, 1H, Ar), 7.82–7.85 (d, 2H, Ar), 12.20 (brs, 1H, -NH). ^13^C-NMR (DMSO-*d*_6_) δ: 14.2, 21.8, 26.0, 37.8, 38.7, 38.9, 39.2, 39.8, 40.1, 40.3, 45.3, 45.7, 53.5, 64.8, 113.2, 123.7, 124.6, 126.3, 130.0, 131.4, 137.0, 147.8, 149.8, 149.9, 153.8, 159.9. IR (KBr pellet): 3147, 3078, 2980, 2937, 2797, 1689, 1602, 1457, 1349, 1289, 1168 cm^−1^.

## 3. Results and Discussion

Chlorosulfonation is a two-stage process: in the first stage, aromatic compound **2** was converted into intermediate product sulfonic acid (**5**), then compound **5** was converted into sulfonyl chloride (compound **3**) in the presence of excess of chlorosulfonic acid. We found that during the chlorosulfonation of compound **2** with chlorosulfonic acid to produce 5-(5-chlorosulfonyl-2-ethoxyphenyl)-1-methyl-3-*n*-propyl-1,6-dihydro-7*H*-pyrazolo[4,3-*d*]pyrimidin-7-one (**3**), about 10% of 5-[2-ethoxy-5-(sulfonyl)phenyl]-1-methyl-3-*n*-propyl-1,6-dihydro-7*H*-pyrazolo[4,3-*d*]pyrimidin-7-one (**5**) left as unreacted product, which was carried into sildenafil as an impurity. It was difficult to separate compound **5** from sildenafil and typically required repeated crystallizations to achieve the desired quality of sildenafil, which was not feasible for any generic pharmaceutical company in terms of cost and yield of the product. In view of this difficulty, we have designed the chlorosulfonation reaction in such a way that compound **2** is reacted with chlorosulfonic acid in the presence of thionyl chloride at room temperature and the reaction is monitored by HPLC until the sulfonic acid derivative, compound **5**, is less than 2%. Addition of thionyl chloride as a chlorinating agent reduced the amount of unreacted sulfonic acid, compound **5**, and provided consistent results. By this simple modification, the overall yield of this process increased to 90% from 70% [3]. We have successfully established this process on a commercial scale.

**Scheme 3:**
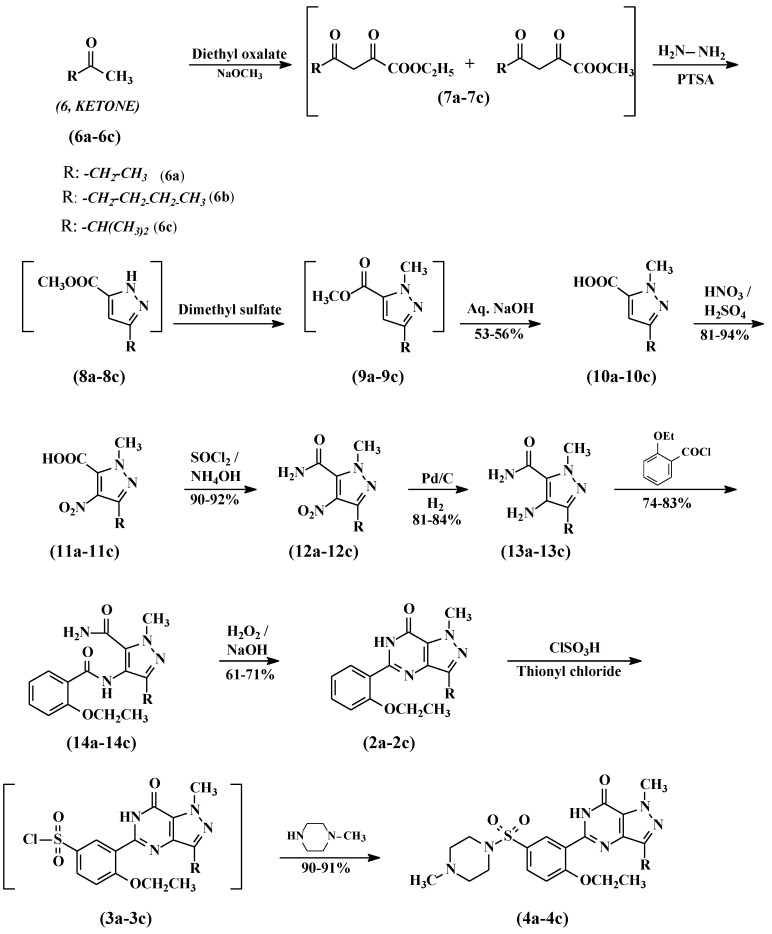
Synthetic route for sildenafil-related compounds.

Several sildenafil analogues and related compounds were reported in the literature [5,6,7,8,9,10,11,12,13,14,15,16,17,18]. In the present work [19], we also report the synthesis of three novel analogues (Scheme 3) of sildenafil, namely (i) 5-[2-ethoxy-5-(4-methylpiperazinylsulfonyl)phenyl]-1-methyl-3-ethyl-1,6-dihydro-7*H*-pyrazolo[4,3-*d*]pyrimidin-7-one (sildenafil ethyl analogue, **4a**); (ii) 5-[2-ethoxy-5-(4-methylpiperazinylsulfonyl)phenyl]-1-methyl-3-*n*-butyl-1,6-dihydro-7*H*-pyrazolo[4,3-*d*]pyrimidin-7-one (sildenafil butyl analogue, **4b**); and (iii) 5-[2-ethoxy-5-(4-methylpiperazinylsulfonyl)phenyl]-1-methyl-3-isopropyl-1,6-dihydro-7*H*-pyrazolo[4,3-*d*]pyrimidin-7-one (sildenafil isopropyl analogue, **4c**) using the improved chlorosulfonation reaction. All of these novel analogues were characterized by using IR, ^1^H-NMR, ^13^C-NMR, and elemental analysis.

## References

[1] Anastasas P.T., Warner J.C. (1988). Green Chemistry: Theory and Practice.

[2] Constable D.J.C., Curzons A.D., Cunningham V.L. (2002). Metrics to ‘green’ chemistry—which are the best?. Green Chem..

[3] Andrew S.B., David B., Nicholas K.T. (1993). Pyrazolopyrimidinone Antianginal Agents. U.S. Patent.

[4] Spryskov A.A., Kuzmina Y.L. (1958). Sulfonation reaction. XLVI. Equilibrium between toluenetrisulfonic acid and its trichloride. Russ. J. Gen. Chem..

[5] Mojzych M., Karczmarzyk Z., Wysocki W., Ceruso M., Supuran C.T., Krystof V., Urbanczyk-Lipkowska Z., Kalicki P. (2015). New approaches to the synthesis of sildenafil analogues and their enzyme inhibitory activity. Bioorg. Med. Chem..

[6] Da Y.F., Huang Y.-L., Mo W.-J. (2014). Synthesis technology of sildenafil analogues. Zhongguo Dangdai Yiyao.

[7] Flores T., Haroldo A., Priviero F.B.M., Teixeira C.E., Perissutti E., Fiorino F., Severino B., Frecentese F., Lorenzetti R., Baracat J.S. (2008). Synthesis and Pharmacological Evaluations of Sildenafil Analogues for Treatment of Erectile Dysfunction. J. Med. Chem..

[8] Ghozlan S.A.S., Badahdah K.O., Abdelhamid I.A. (2007). An easy synthesis of 5-functionally substituted ethyl 4-amino-1-aryl-pyrazolo-3-carboxylates: Interesting precursorsto sildenafil analogues. Beilstein J. Org. Chem..

[9] Patel P., Joshi N. (2012). Process for Preparation of 5-(2-Ethoxy-5-((4-methylpiperazin-1-yl)sulfonyl) phenyl)-3-isobutyl-1-methyl-1H-pyrazolo[4,3-*d*]pyrimidin-7-(6H)-one (Sildenafil Citrate Impurity). Indian Patent.

[10] Patel P.V., Joshi N., Panchal D.P. (2012). Process for preparation of 5-(2-ethoxy-5-((4-methylpiperazin-1-yl)sulfonyl)phenyl)-3-isobutyl-1-methyl-1H-pyrazolo[4,3-*d*]pyrimidin-7(6H)-one (sildenafil citrate impurity). Heterocycl. Lett..

[11] Saravanan M., Satyanarayana B., Pratapreddy P. (2011). Synthesis and characterization of potential impurities of sildenafil. Chem. Biol. Interface.

[12] Kumar I.V.S., Ramanjaneyulu G.S., Bindu V.H. (2011). Synthesis of Sildenafil citrate and process related impurities. Lett. Org. Chem..

[13] Zhang M., Xia Z., Ma Y., Zang L., Chen Z. (2013). Synthesis of related substances of sildenafil. Zhongguo Yiyao Gongye Zazhi.

[14] Julie V., Veronique G., Stephane B., Chantal Z., Robert M., Myriam M.M. (2012). Identification of a novel sildenafil analogue in an adulterated herbal supplement. J. Pharm. Biomed. Anal..

[15] Ge X., Li L., Koh H.L., Low M.Y. (2011). Identification of a new sildenafil analogue in a health supplement. J. Pharm. Biomed. Anal..

[16] Kim D.K., Lee N., Lee J.Y., Ryu D.H., Kim J.S., Lee S.H., Choi J.Y., Chang K., Kim Y.W., Im G.J. (2001). Synthesis and phosphodiesterase 5 inhibitory activity of novel phenyl ring modified sildenafil analogues. Bioorg. Med. Chem..

[17] Daraghmeh N., Al-Omari M., Badwan A.A., Jaber A.M.Y. (2001). Determination of sildenafil citrate and related substances in the commercial products and tablet dosage form using HPLC. J. Pharm. Biomed. Anal..

[18] Iwona W., Maciej P., Zdzislaw C. (2005). ^1^H, ^13^C, ^15^N NMR analysis of sildenafil and citrate (viagra) in solution, solid state and pharmaceutical dosage forms. J. Pharm. Biomed. Anal..

[19] Raghava Reddy A.V., Srinivas G., Shantankumar K., Joseph Prabahar K., Sivakumaran M. (2013). An Improved Process for the Preparation of Sildenafil Citrate. Indian Patent.

